# Space use and genetic structure do not maintain color polymorphism in a species with alternative behavioral strategies

**DOI:** 10.1002/ece3.4729

**Published:** 2018-12-26

**Authors:** Madeleine St Clair Yewers, Devi Stuart‐Fox, Claire Alice McLean

**Affiliations:** ^1^ School of BioSciences The University of Melbourne Melbourne Victoria Australia; ^2^ Sciences Department, Museum Victoria Carlton Gardens Victoria Australia

**Keywords:** color polymorphism, *Ctenophorus decresii*, lizard, microsatellites, territory size

## Abstract

Space use including territoriality and spatial arrangement within a population can reveal important information on the nature, dynamics, and evolutionary maintenance of alternative strategies in color polymorphic species. Despite the prevalence of color polymorphic species as model systems in evolutionary biology, the interaction between space use and genetic structuring of morphs within populations has rarely been examined. Here, we assess the spatial and genetic structure of male throat color morphs within a population of the tawny dragon lizard, *Ctenophorus decresii*. Male color morphs do not differ in morphology but differ in aggressive and antipredator behaviors as well as androgen levels. Despite these behavioral and endocrine differences, we find that color morphs do not differ in territory size, with their spatial arrangement being essentially random with respect to each other. There were no differences in genetic diversity or relatedness between morphs; however, there was significant, albeit weak, genetic differentiation between morphs, which was unrelated to geographic distance between individuals. Our results indicate potential weak barriers to gene flow between some morphs, potentially due to nonrandom pre‐ or postcopulatory mate choice or postzygotic genetic incompatibilities. However, space use, spatial structure, and nonrandom mating do not appear to be primary mechanisms maintaining color polymorphism in this system, highlighting the complexity and variation in alternative strategies associated with color polymorphism.

## 1 INTRODUCTION

In color polymorphic species, discrete, heritable morphs within a population often differ in multiple traits (e.g., morphology, physiology, life history, and behaviors), which in combination affect fitness to form alternative strategies (Fisher, 1930; Roulin, 2004; Sinervo & Lively, 1996). The fitness of morphs can depend on the frequency of interactions between individuals with different strategies (Bleay, Comendant, & Sinervo, 2007; Pryke, Astheimer, Buttemer, & Griffith, 2007), generating frequency‐dependent selection (Dijkstra et al., 2010; Takahashi, Yoshimura, Morita, & Watanabe, 2010). Consequently, space use can reveal important information on the nature and dynamics of alternative strategies. Morphs can be nomadic or territorial (Hurtado‐Gonzales & Uy, 2009; Thompson & Moore, 1991) and variations in the size and environmental qualities of territories can further reflect alternative strategies (Lattanzio & Miles, 2014; Zamudio & Sinervo, 2000) including monopolization of resources and/or mates (Brown, 1964; Kaufmann, 1983). Nonrandom spatial arrangement of morphs within a population can have a strong ecological component (Ahnesjö & Forsman, 2006; Forsman, Ahnesjö, Caesar, & Karlsson, 2008; Munday, Eyre, & Jones, 2003); however, when morphs display alternative behavioral or reproductive strategies, the social environment can be equally if not more important than ecological factors in determining the spatial arrangement of individuals (Moore, Brodie, & Wolf, 1997). Spatial clustering of certain morph types often results when the social environment affects the relative success of each strategy (Formica & Tuttle, 2009; Sinervo & Clobert, 2003). For example, in the white‐throated sparrow *Zonotrichia albicollis*, the promiscuous, aggressive white male morph settles in high‐density areas where the potential to encounter mates is highest, whereas the mate guarding tan male morph is found in low‐density areas where the risk of intruders is lower (Formica, Gonser, Ramsay, & Tuttle, 2004). Despite the importance of space use in maintaining polymorphism, detailed data on territory size, overlap, and spatial arrangement exist for a limited number of polymorphic systems (but see Calsbeek & Sinervo, 2002, Olsson, Wapstra, Healey, Schwartz, & Uller, 2008, Paterson & Blouin‐Demers, 2018, Sinervo, Chaine, et al., 2006b).

Morphs can also spatially segregate based on genetic similarity. Male color morphs of the side‐blotched lizard, *Uta stansburiana*, are located nonrandomly, with genetically similar but unrelated blue males settling in close proximity, increasing their fitness compared to blue males that do not have like‐morph neighbors. By contrast, behaviorally dominant, genetically similar orange males are hyperdispersed and decrease each other's fitness when neighbors. Although blue male neighbors are not kin, they nevertheless cooperate, displaying greenbeard altruism where cooperators can identify individuals with similar alleles to themselves (Sinervo & Clobert, 2003; Sinervo, Chaine, et al., 2006b). Genetic differences between morphs can also be manifested as differences in dispersal tendencies (Sinervo, Calsbeek, et al., 2006a). Although dispersal can be phenotypically plastic and can depend on the social and ecological environment (Bowler & Benton, 2005), it is a major driver of genetic structure within populations (Sugg, Chesser, Dobson, & Hoogland, 1996). Genetic differences between morphs are often reinforced by nonrandom mating (Bleay & Sinervo, 2007; Houtman & Falls, 1994; Huyghe et al., 2010), in particular, assortative mating may further restrict gene flow and, under restricted conditions (e.g., ecological constraints), can lead to reproductive isolation (Elmer, Lehtonen, & Meyer, 2009; Jiang, Bolnick, & Kirkpatrick, 2013; Kirkpatrick & Ravigné, 2002). Thus, quantifying genetic similarities within morphs and genetic differentiation between morphs is essential to understand processes maintaining color polymorphism or promoting speciation (Gray & McKinnon, 2007; McKinnon & Pierotti, 2010).

Here, we assess the spatial and genetic structure of color morphs within a population of the tawny dragon lizard, *Ctenophorus decresii *(Duméril & Bibron, 1837). *Ctenophorus decresii *males are polymorphic for throat color, with four discrete throat color morphs: orange, gray, yellow, and orange‐yellow (a yellow throat with a central orange patch; Teasdale, Stevens, & Stuart‐Fox, 2013) which is displayed prominently during intraspecific communication (Gibbons, 1979; Osborne, 2005; Stuart‐Fox & Johnston, 2005) and *C. decresii *can visually distinguish (Yewers et al., 2015). Conversely, all females have uniformly cream colored throats, but can be induced to express the same set of throat color morphs through artificial elevation of testosterone levels (Rankin & Stuart‐Fox, 2015). Color morphs are fixed from sexually maturity (Rankin & Stuart‐Fox, 2015; Teasdale et al., 2013), throat color is autosomally inherited, and the proportion of yellow or orange on the throat is highly heritable (Rankin, McLean, Kemp, & Stuart‐Fox, 2016).

Males defend their territories using complex displays, involving extending and lowering the throat, head‐bobs, hind‐leg push‐ups, and lateral compressions (Gibbons, 1979; Osborne, 2005; Stuart‐Fox & Johnston, 2005). Low rates of multiple paternity and close proximity between fathers and mothers suggest successful territoriality in this species (Hacking, Stuart‐Fox, & Gardner, 2018). Color morphs do not differ in morphology but differ in aggression toward territory intruders, antipredator behaviors, and hormonal profiles (Yewers, Jessop, & Stuart‐Fox, 2017; Yewers, Pryke, & Stuart‐Fox, 2016). The orange morph shows consistent high levels of aggression to conspecifics, the gray morph is consistently less aggressive, and the aggression of orange‐yellow and yellow morphs depends on the color of their competitor. The gray morph is not only the least aggressive but is also the least bold toward a potential predator compared to the other morphs, which all have similar boldness (Yewers et al., 2016). Furthermore, the gray and yellow morphs have low androgen levels immediately after capture compared to high levels of androgen in the orange and orange‐yellow morphs. Following acute stress, androgen levels of the gray and yellow morphs increase, while androgen levels of the orange and orange‐yellow morphs remain high (Yewers et al., 2017). Combined, behavioral responses and hormonal profiles indicate that the gray morph employs a cautious strategy, while the orange morph is most likely dominant. Little is known about dispersal in this species but in the congeneric *Ctenophorus ornatus*, males and females do not differ in dispersal patterns (LeBas, 2002). However, the role of space use, including the likelihood of interactions between morphs, and genetic structure in defining morph‐specific strategies and in maintaining the polymorphism remains unclear.

We investigated whether there are differences in the spatial arrangement and territory size of the four color morphs of *C. decresii*. A previous study found no difference in microhabitat use, and thus territory quality among morphs (Teasdale et al., 2013); however, it is not known whether morphs differ in territory size. Among color polymorphic lizards, there is no consistent relationship between dominance of morphs in staged contests and territory size. For example, in side‐blotched lizards (*Uta stansburiana*), dominant orange males control and defend the largest territories (Sinervo & Lively, 1996; Zamudio & Sinervo, 2000; Sinervo et al. 2000, 2006; Calsbeek & Sinervo, 2002); whereas in tree lizards (*Urosuarus ornatus*), dominant blue males defend small, defined territories compared to males of the other morphs (Lattanzio & Miles, 2014; Thompson & Moore, 1991), and in painted dragons (*Ctenophorus pictus*), the territory size of dominant red males is no bigger than that of yellow males (Olsson et al., 2008). Furthermore, in tree lizards, the least dominant orange morph may be territorial or nomadic depending on resources, which vary between years (Moore, Hews, & Knapp, 1998; Paterson & Blouin‐Demers, 2018). In addition to territory size, we assessed the spatial arrangement of individuals to test whether males of each morph are located in the population in a nonrandom way, indicative of dispersal behaviors or population dynamics. For example, due to differences in behavior, it may be advantageous for some combinations of morphs to be neighbors (Sinervo & Clobert, 2003) or certain morphs may disperse further than others (Sinervo, Calsbeek, et al., 2006a). We also compared the genetic similarity and relatedness of individuals within and among morphs. In the absence of spatial clustering of morphs, high relatedness and greater genetic similarity within morphs may indicate behavioral or genetic barriers to gene flow (i.e., nonrandom or assortative mating; Pérez I De Lanuza, Font, & Carazo, 2013).

## 2 METHODS

### 2.1 Study species

**Figure 1:**
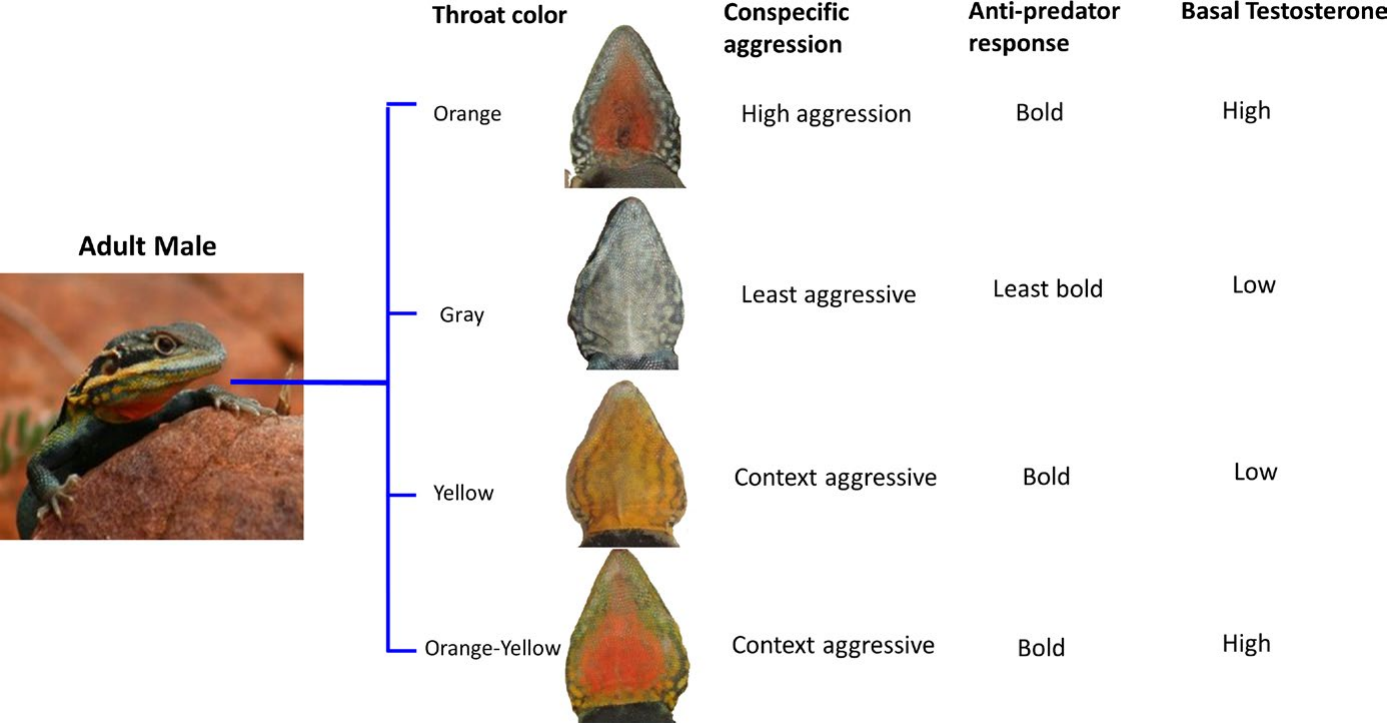
Male throat color morphs of the tawny dragon lizard, *Ctenophorus decresii *(from top: orange, gray, yellow, and orange‐yellow) and correlated behavioral and physiological traits. Image adapted from Yewers et al (2017)

The tawny dragon lizard, *Ctenophorus decresii*, is a small, sexually dimorphic agamid lizard found on rocky outcrops of the southern Flinders Ranges, Mt Lofty Ranges, and Kangaroo Island in South Australia (Houston, 1974). Males vary in throat color both within and among populations (Houston & Hutchinson, 1998; McLean, Stuart‐Fox, & Moussalli, 2014). In populations in the Flinders Ranges, there are four distinct male throat color morphs: orange, yellow, gray, and orange‐yellow, which can be objectively classified independently of the human visual system (Teasdale et al., 2013; Figure 1). Orange males have a variably sized orange patch on a gray/cream reticulated background; yellow males have a variably sized yellow patch on a gray/cream reticulated background; orange‐yellow males have variably sized orange patch on a yellow background; and gray males have a gray/cream reticulated throat with no yellow or orange present. Yellow and orange coloration is produced by a combination of carotenoids obtained from the diet and pteridines produced by specialized organelles within the xanthophore pigment cells (McLean, Lutz, Rankin, Stuart‐Fox, & Moussalli, 2017).

### 2.2 Study site and population

We studied a wild population of tawny dragon lizards at the Yourambulla Caves Historic Reserve in the Flinders Ranges, South Australia, Australia (138º 37’ E 31º 95’ S), during two breeding seasons between October and December 2011 and 2012. The reserve is located in a semi‐arid region and lizards are found in rocky areas interspersed with ground cover, casuarinas, cypress pines, and low‐lying shrubs. We captured 98 male lizards in 2011 and 92 male lizards in 2012, either by hand or by noosing (using a telescopic pole and noose made of fishing line). To ensure that males were sexually mature, and thus exhibiting adult throat coloration, we only included males greater than 76‐mm snout‐vent length in this study (considered adults when >65 mm; Gibbons, 1979). For each male caught, we took a blood sample (100–200 μl) by venipuncture from the *sinus angularis *accessed from the corner of the mouth and collected with 100‐μl heparinized capillary tubes (Jessop, Chan, & Stuart‐Fox, 2009; Olsson, Wapstra, Madsen, & Silverin, 2000). We collected red blood cells from whole blood by centrifugation which were stored at −20°C for subsequent molecular analysis. We permanently marked individuals using subcutaneous colored elastomer implants (Northwest Marine Technology). Elastomer implants are commonly used in fish and amphibians and do not cause permanent injury compared to marking by toe clipping (Calsbeek, Bonneaud, & Smith, 2008; Nauwelaerts, Coeck, & Aerts, 2000). For visual recognition, we wrote a temporary unique number on the back of each lizard using a nontoxic xylene‐free Pilot paint pen (Pilot Corporation, Tokyo, Japan).

### 2.3 Territory size

**Figure 2:**
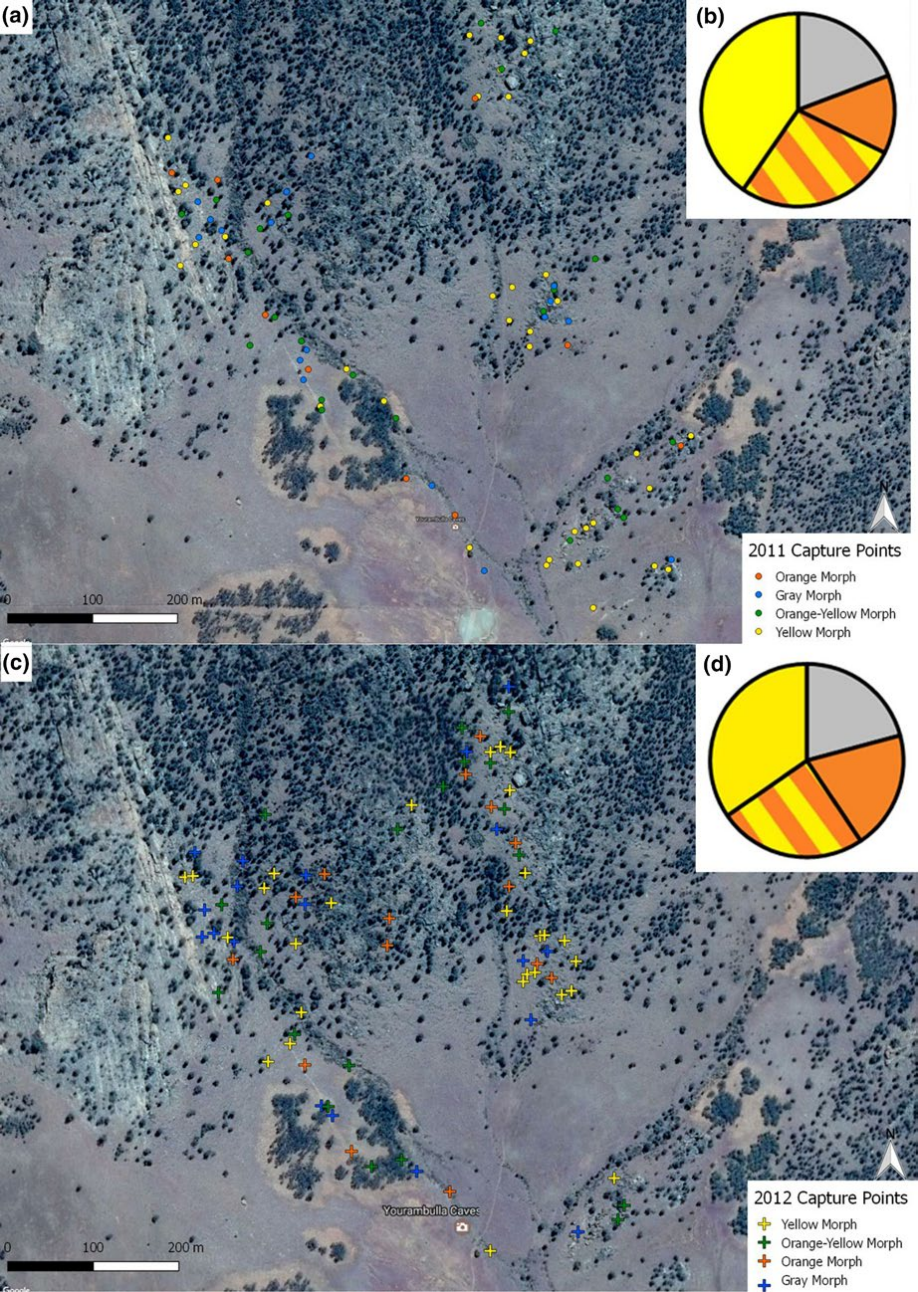
(a) Capture points of all male adult lizards from the 2011 field season. Morphs are represented with colored points. (b) Percentage of each color morph in the population caught in 2011. Yellow represents the yellow morph, grey represents the grey morph, orange represents the orange morph and the orange and yellow bars represents the orange‐yellow morph. (c) Capture points of all male adult lizards from the 2012 field season. Morphs are represented with colored crosses. (d) Percentage of each color morph in the population caught in 2012. Yellow represents the yellow morph, grey represents the grey morph, orange represents the orange morph and the orange and yellow bars represents the orange‐yellow morph

To determine territory size, we recorded the spatial coordinates with a GPS (Garmin GPSMAP® 76CSx, Garmin Ltd., USA) within 4‐meter accuracy for each capture point and resighting of a paint‐marked adult male using binoculars to assess the identification number (Figure 2). We did not combine sightings across years so that estimated territory sizes were year‐specific. We estimated territory size using 95% fixed kernel range sizes calculated using least squares cross‐validation, and only used territory estimates where areas did not increase following incremental analysis and therefore leveled out asymptotically (Ranges 9; Anatrack Ltd., Wareham, UK). This resulted in a median number of eight sightings (range of 4–22 sightings) per territory estimate for 31 adult male individuals: nine orange, eight orange‐yellow, seven yellow, and seven gray. Territory size estimates were log_10_‐transformed to meet assumptions of normality.

For territory size estimates, we targeted certain individuals to ensure sufficient sample sizes for each color morph. It was not possible to quantify the territory boundaries of all individuals in the population and therefore the exact proportion of territory overlap between neighboring individuals of each color morph. However, we estimated potential encounters between neighbors by placing a buffer of average territory size (buffer radii = 14.16 m around each capture point for each male adult lizard encountered, including those for which we did not estimate territory size or resight (QGIS 3.2; Open Source Geospatial Foundation Project. http://qgis.osgeo.org). Therefore, the sample sizes for encounter rates were a lot larger than for territory size estimates (sample sizes: gray = 26, orange = 20, orange‐yellow = 34, yellow = 45). For each focal male, we counted how many times its buffer overlapped with a buffer of another male adult lizard to give the total number of overlaps per focal male. We noted the color morph of the focal territory holder and the color morph of the individual that overlapped with the territory of the focal male for each overlap in buffers and tallied the number of times each possible combination of color morphs overlapped in territory. To further investigate differences between morphs in territoriality, we compared the number of times individuals were resighted in the regularly surveyed areas of the study site. We also compared the proportion of new sightings over time between color morphs. We were unable to confidently quantify the number of females present in each male's territory because females are cryptic and do not defend territories from prominent perch sites, so they are less commonly seen.

### 2.4 Statistical analysis

We tested for differences between the color morphs in territory size and number of resightings using general linear models (Proc GLM; SAS v.9.2) with color morph, year, and their interaction as fixed factors. Territory size was not estimated for the same individual in the 2 years.

To first confirm that we had caught the majority of adult male lizards at the field site in each year, we performed a chi‐squared test (*χ* ^2^; Proc FREQ; SAS v.9.2) for each year, testing whether there were differences between color morphs in the proportion of captures over four consecutive time periods. To detect differences between morphs in the number of possible encounters with neighbors, we used a generalized linear model with Poisson distribution and log link function (Proc GENMOD; SAS v.9.2). The total number of potential overlaps for each male (based on territory “buffers”) was the response variable, and focal male morph, year, and their interaction were included as fixed effects in the model. Furthermore, we conducted a chi‐squared test to test for differences in the number of observed and expected pairwise combinations of color morphs resulting from approximate territory overlap. Note that calculation of the expected number of overlaps in the contingency table accounts for differences in the relative frequency of each morph in the population.

We used the program G*Power (Faul, Erdfelder, Buchner, & Lang, 2009; Faul, Erdfelder, Lang, & Buchner, 2007) to determine the effect size we could detect given our sample size. Our sample size had power of 0.8 (which is considered an appropriate level of statistical power; Cohen, 1988) to detect territory size differences between morphs with effect size, Cohen's *d* = 0.638. This effect size is at the lower end of the range of effect sizes detected by other studies comparing territory size between groups and/or sexes (0.58 and 7.09), which were calculated from published work (Awata & Kohda, 2005; Braun, 2016; Costa et al., 2011; Kjellander et al., 2004; Morrison, Keogh, & Scott, 2002; Schradin, Scantlebury, Pillay, & Kӧnig, 2009) using the method of Thalheimer & Cook (2002). This suggests that we had sufficient power to detect even subtle biologically meaningful differences between territory sizes of morphs.

### 2.5 Microsatellite genotyping

To assess the genetic diversity within and among color morphs, we genotyped 119 adult males (55 caught in 2011 and 64 caught in 2012 for which we had viable blood samples) for eight microsatellite loci. If a male was caught in both years, we only used one sample per individual. We extracted DNA from red blood cells using proteinase‐K and a GenCatch (TM) Blood & Tissue Genomic Mini‐Prep Kit (Epoch Life Sciences, Sugar Land, TX, USA). We used published polymerase chain reaction (PCR) protocols (McLean et al., 2014) for six microsatellite loci developed specifically for *C. decresii *(Ctde03, Ctde05, Ctde08, Ctde12, Ctde21, Ctde45; McLean et al., 2014) and two microsatellite loci for the congeneric *Ctenophorus pictus* (CP10, CP11; Schwartz, Warner, Beheregaray, & Olsson, 2007). Fragment visualization was performed by Macrogen (Korea) using a GeneScan‐500 LIZ size standard (Applied Biosystems, Foster City, CA, USA) and we called fragment sizes using Peak Scanner 2 ver. 2.0 (Applied Biosystems). We assigned alleles using automated binning in Flexibin software (Amos et al., 2007) with manual edits. All loci were checked for the presence of null alleles using Microchecker ver. 2.2.3 (Van Oosterhout, Hutchinson, Wills, & Shipley, 2004) and tested for linkage disequilibria and Hardy–Weinberg equilibria within and across color morphs using GENEPOP ver. 4.2 (Raymond & Rousset, 1995; Rousset, 2008).

### 2.6 Genetic structure within and among morphs

We first assessed allele frequencies within morphs across years through a pairwise comparison of *F*_ST_ using GenAlEx. There was no difference between years (*F*_ST_ = 0.040, *p* = 0.298); therefore, we combined data across years. We assessed genetic diversity for the whole population and within morphs by calculating observed heterozygosity, expected heterozygosity, and the inbreeding coefficient (*F*_IS_) for each locus in GenAlEx ver. 6.5 (Peakall & Smouse, 2006). We also calculated allelic richness for each locus, with rarefaction correction for uneven sample size, in HP‐Rare (Kalinowski, 2005). Differences in genetic diversity (*F*_IS_, observed and expected heterozygosity and allelic richness) among morphs were tested using a one‐way ANOVA and Tukey's post hoc tests (Proc GLM; SAS v.9.2). Additionally, we used the Queller and Goodnight (1989) coefficient of relatedness estimator in GenAlEx to determine the mean relatedness of each morph on a scale of −0 (not related) to ±1 (clones or identical twins), with negative values resulting when two individuals differ from the population mean in different directions.

Genetic divergence between color morphs may indicate nonrandom mating processes, for example, assortative mating. We measured genetic differentiation between morphs by calculating the Fixation Index (*F*_ST_) for the whole population and pairwise *F*_ST_ between morphs in FreeNa which performs analyses with and without null alleles (Chapuis & Estoup, 2007). Null allele frequencies are estimated through the expectation–maximization algorithm (Dempster, Laird, & Rubin, 1977). We used GenAlEx to conduct an analysis of molecular variance (AMOVA) to calculate the proportion of genetic differentiation within versus among morphs. We assessed the similarity of allelic composition of morphs with factorial correspondence analysis (FCA) in GENETIX ver. 4.05 (Belkhir, Borsa, Chikhi, Raufaste, & Bonhomme, 1996‐2004). We conducted a multivariate analysis of variance to test for differences in FCA factors between the color morphs (PROC GLM; SAS v.9.2) with a significant difference in factors indicating genetic differentiation.

Finally, we investigated whether the spatial arrangement of individuals within the population was influenced by genetic distance. To do this, we performed Mantel tests of matrix correspondence in GenAlEx with a linear genetic distance matrix of individual‐by‐individual pairwise comparisons as the independent variable following the established protocol of Smouse, Long, and Sokal (1986) and geographic distances generated by calculating the Euclidean distance between the centroid of territories as the dependent variable. This was performed for the whole population and within each color morph to test whether the genetic distribution of morphs within a population is affected by geographic distance, which could be due to dispersal behavior based on genetic similarity. We ran all randomization procedures for all analyses with 10^4^ iterations and applied false discovery rate correction for all multiple testing (Benjamini & Hochberg, 1995).

## 3 RESULTS

### 3.1 Spatial arrangement

**Figure 3:**
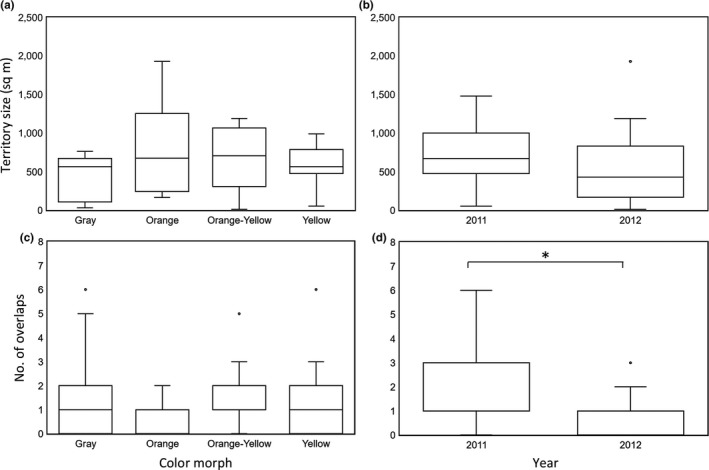
(a) Territory size (log square meters; mean ± *SE*) of each color morph: gray (*n* = 7), orange (*n* = 9), orange‐yellow (*n* = 8), and yellow (*n* = 7). (b) Territory overlap (number of overlaps in approximate territories; mean ± *SE*) of color morphs: gray (*n* = 26), orange (*n* = 20), orange‐yellow (*n* = 34), and yellow (*n* = 45). (c) Territory overlap (number of overlaps in approximate territories; mean ± *SE*) during 2011 (*n* = 69) and 2012 (*n* = 56). d) Territory overlap (number of overlaps in approximate territories; mean+/‐ SE) during 2011 (*n* = 54) and 2012 (*n* = 69). Morphs do not differ in territory size and degree of territory overlaps but territory overlap differs between years sampled

There was no difference in the number of resightings between morphs (*F*_3,52_ = 1.24, *p* = 0.31) or the number of resightings per year (*F*_1,52_ = 0.64, *p* = 0.43). There was no difference in territory size between morphs (*F*_3,23_ = 0.75, *p* = 0.53; Figure 3a), year (*F*_1,23_ = 3.16, *p* = 0.09), or their interaction (*F*_3,23_ = 2.07, *p* = 0.13). To maximize statistical power to detect territory size differences between morphs, and because year was not significant, we reran the model with morph as the only fixed factor and it was not statistically significant (*F*_3,27_ = 0.54, *p* = 0.66; Figure 3a).

There was also no difference in the number of potential encounters with neighbors between morphs ($\chi_{\text{3}}^{\text{2}}$ = 3.06, *p* = 0.38, *n* = 125; Figure 3c) and no interaction between morph and year ($\chi_{\text{3}}^{\text{2}}$ = 2.57, *p* = 0.46). However, the number of potential encounters based on territory overlap was greater in 2011 than 2012 (territory overlap: $\chi_{\text{1}}^{\text{2}}$ = 10.03, *p* = 0.0015; Figure 3d).

There was no difference in the number of new captures over four consecutive time periods between color morphs in 2011 ($\chi_{\text{9}}^{\text{2}}$ = 13.89, *p* = 0.13), or 2012 ($\chi_{\text{9}}^{\text{2}}$ = 7.68, *p* = 0.57) so we are confident in our sampling and the representation of all morphs across the entire breeding season. There was also no difference in the likelihood of certain color morphs neighboring one another based on the overlap of their estimated territories ($\chi_{\text{9}}^{\text{2}}$ = 8.13, *p* = 0.52).

### 3.2 Microsatellite analysis

Four loci (Ctde03, Ctde21, Ctde45, and CP10) deviated from Hardy–Weinberg Equilibrium (HWE) and Microchecker suggested evidence of null alleles across all samples at these loci (Table 1). We found that no locus consistently deviated from HWE when we examined color morphs separately (Table 2). There was no difference in statistical significance in the presence or absence of null alleles (Table 3; Supporting Information Appendix S1: Table S1); therefore, we contain analysis for all data including possible null alleles. Across all samples, two pairs of loci showed linkage disequilibria (Ctde08 and Ctde45, Ctde12 and CP10); however, only one pair (Ctde08 and Ctde45) showed consistent linkage disequilibrium within all morphs (Supporting Information Appendix S1: Table S2). Therefore, we compared all analyses with and without Ctde08 or Ctde45, which did not qualitatively change results. Given that loci did not consistently deviate from HWE across morphs, there was no difference in results including loci in linkage disequilibrium and there was no difference in data where null alleles were present or absent, we report results from analyses incorporating all loci.

**Table 1:** Genetic diversity statistics for the whole population, all morphs combined

Locus	*N*	*N*_a_	*A*_r_	*H*_o_	*H*_e_	*F*_IS_	*p*
Ctde03	92	15	5.862	0.737	0.889	0.105	***<0.001***
Ctde05	119	15	6.308	0.96	0.918	0.03	0.12
Ctde08	119	12.5	5.766	0.72	0.854	0.094	0.54
Ctde12	119	13	5.8	0.92	0.901	−0.019	0.695
Ctde21	106	9	5.107	0.619	0.856	0.177	***<0.001***
Ctde45	110	10	5.458	0.591	0.805	0.186	***0.014***
CP10	116	13.5	5.972	0.708	0.884	0.101	***<0.001***
CP11	119	4	2.842	0.574	0.562	0.052	0.506

Note

*N* sample size at each locus*, N*_a_ number of alleles, *A*_r_ allelic richness, Ho observed heterozygosity, He expected heterozygosity, *F*_IS_ inbreeding coefficient, and *p *deviation from Hardy–Weinberg equilibrium. Significant values in bold remained significant after false discovery rate correction.

**Table 2:** *F*_IS_ values and deviations from Hardy–Weinberg equilibrium (HWE) as tested by a chi‐squared test for each color morph at each locus

Morph	Gray (*N* = 24)		Orange (*N* = 21)		OY (*N* = 33)		Yellow (*N* = 36)	
Locus	*F*_IS_	*p*	*F*_IS_	*p*	*F*_IS_	P	*F*_IS_	*p*
Ctde03	0.198	**0.020**	0.035	0.642	0.28	**<0.001**	0.078	0.236
Ctde05	−0.026	0.779	0.197	**0.012**	0.026	0.262	0.001	0.397
Ctde08	0.176	0.033	0.1	0.252	0.032	0.943	0.139	0.028
Ctde12	−0.001	0.673	0.026	0.785	−0.005	0.899	−0.023	0.420
Ctde21	0.299	**0.002**	0.061	0.469	0.245	**0.001**	0.281	0.009
Ctde45	0.287	**0.011**	0.297	**0.007**	0.066	0.41	0.176	0.073
CP10	0.219	**0.011**	0.106	0.239	0.139	**<0.001**	0.116	0.036
CP11	0.096	0.793	−0.107	0.733	0.209	0.114	0.087	0.657
Overall	0.149	<0.001	0.099	0.033	0.115	<0.001	0.104	0.003

Note

The orange‐yellow morph has been abbreviated to OY. Significant deviations from HWE in bold remained significant after false discovery rate correction.

**Table 3:** Genetic differentiation between color morphs of *C. decresii *generated from multilocus genotypes (*n* = 8 microsatellite loci), with pairwise* F*_IS_ values and *p*‐values. Comparison

	*F*_IS_	*p*
Gray and orange	0.004	0.370
Gray and orange‐yellow	0.013	0.081
Gray and yellow	0.004	0.336
Orange and orange‐yellow	0.002	0.529
Orange and yellow	0.005	0.062
Orange‐yellow and yellow	<−0.001	1.02
Overall including estimated null alleles	0.004	0.945
Overall without estimated null alleles	0.005	0.581

Note

No pairwise *F*_IS_ values remained significant after false discovery rate correction.

### 3.3 Is there restricted gene flow between color morphs?

The *F*_IS_ across all samples was mainly positive (Table 1), indicating that individuals are more related than expected under random mating. However, when we considered morphs independently, we found a mix of positive and negative *F*_IS_ values (Table 2). Color morphs did not differ in their *F*_IS_ (*F*_3, 28_ = 0.51, *p* = 0.68), observed heterozygosity (*H*_o_; *F*_3,28_ = 0.15, *p* = 0.93), expected heterozygosity (*H*_e_; *F*_3,28_ = 0.02, *p* = 0.99), or allelic richness (*A*_r_; *F*_3,28_ = 0.03, *p* = 0.99), and measures of relatedness for each year were similarly low for all color morphs (Supporting Information Appendix S1: Figure S1).

We detected no genetic divergence between the color morphs at the population level or in pairwise comparisons between morphs (all *p* > 0.05; Table 3). AMOVA revealed that the majority of genetic differentiation was within morphs (99%), with only 1% of the genetic diversity observed among morphs (*F*_ST_ = 0.005, *p* = 0.006).

**Figure 4:**
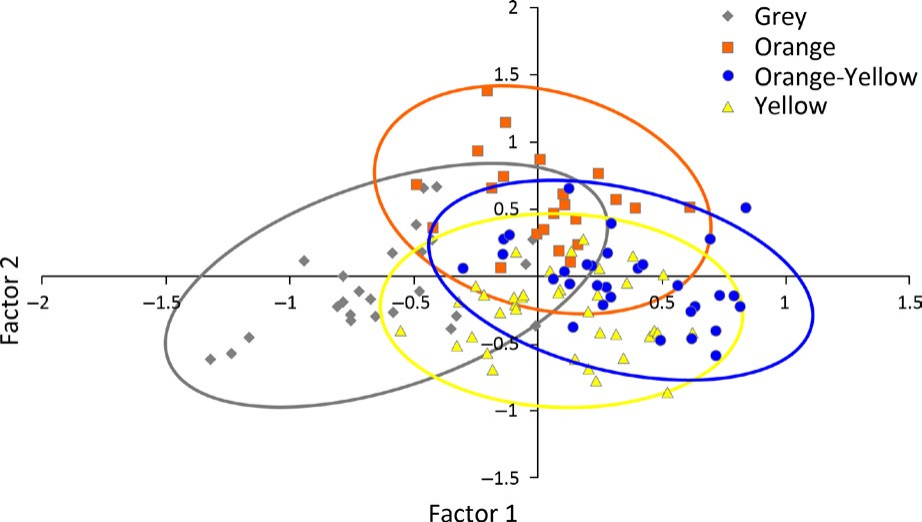
The relationship between Factors 1 and 2 from a factorial correspondence analysis (FCA using microsatellite allele frequencies constructed in Genetix v. 4.05; Belkhir, et al., 1996‐2004). Color morphs form distinct groupings despite substantial overlap (*N* = 119).

The first two factors from the FCA accounted for 44.5% and 31.9% of variation and were able to discriminate color morphs (Wilks’ λ = 0.224, *F*_6,228_ = 42.25, *p* < 0.0001) despite substantial overlap. The plot (Figure 4) shows separation between the gray and orange‐yellow morphs along the *x*‐axis (Factor 1).

### 3.4 Does the spatial arrangement of morphs reflect their genetic structure?

Among morphs, there was no correlation between geographic distance and genetic structure of individuals in either year (Mantel test; 2011: *n* = 53, *r = *0.012, *p* = 0.450; 2012: *n* = 61, *r* = −0.002, *p* = 0.530); that is, mating is random across the space sampled. More related individuals were not more spatially clustered or dispersed than less related individuals. Likewise, when we considered geographic and genetic distance within each morph, there was no evidence for correlation indicating that differentiation of color morphs was not due to their spatial arrangement in either year and that morphs do not differ in dispersal distance (Supporting Information Appendix S1: Table S3).

## 4 DISCUSSION

The spatial and genetic structure of morphs within populations is informative for understanding processes maintaining polymorphisms. Our study on *C. decresii* suggests that all morphs have similar space use, as there were no differences in territory size, capture rates throughout the season, propensity to be resighted or degree of overlap with neighbors. Furthermore, there was no spatial clustering of morphs, with the spatial arrangement of morphs with respect to each other being essentially random. Despite a lack of spatial clustering, we found significant albeit minor, genetic differentiation between morphs and FCA allelic composition. There were no differences in genetic diversity (*F*_IS_, *F*_ST_ *H*_o_, *H*_e_, or Ar measures) or relatedness between morphs and no relationship between geographic and genetic distance, suggesting that overall dispersal distances are likely greater than the fine spatial scale of this study. Thus, the genetic differentiation we detected between morphs cannot be explained by the spatial arrangement of morphs within the population.

We expected that the behavioral differences between morphs would be reflected by differences in space use. However, despite clear behavioral strategies tied to differences in androgen levels (Yewers, 2016), all morphs appear to be territorial and have similar territory sizes. Even though the orange morph is the most aggressive with the highest levels of androgen, it is no larger than other morphs (Teasdale et al., 2013), nor does it have a greater bite force (Yewers et al., 2017). The presence or absence of territoriality or differences in territory size often correlates with reproductive strategy (Hogan‐Warburg, 1966; Taborsky, 2001; Thompson & Moore, 1991; Zamudio & Sinervo, 2000) but this is not always the case (Formica et al., 2004). Similarly to *C. decresii*, in the congeneric polymorphic painted dragon, *Ctenophorus pictus*, male color morphs do not differ in their space use or morphology (Healey & Olsson, 2008; Olsson et al., 2007). Instead, *C. pictus* morphs maintain morph‐specific reproductive strategies through postcopulatory mechanisms coupled with differences in dominance (Healey, Uller, & Olsson, 2007; Olsson, Schwartz, Uller, & Healey, 2009). Indeed, *C. decresii *morphs may differ in behavioral strategies to access and defend females and/or employ postcopulatory reproductive strategies to secure paternity. Further studies into the temporal and spatial reproductive success of male morphs would extend our understanding of the complex role of morph‐specific alternative strategies in this species.

In addition to the lack of differences in territory size, there were no differences in the spatial arrangement of morphs in relation to other morphs or genetic relatedness. Therefore, it is unlikely that the arrangement of male morphs socially restricts the relative success of morph‐specific behavioral strategies, as it does in some other color polymorphic species (Formica et al., 2004; Vercken, Sinervo, & Clobert, 2012), which can result in spatial clustering of morphs (Sinervo & Clobert, 2003). Indeed, although the number of overlaps in territory differed between years, the social environment was consistent in each year with similar density and frequencies of male morphs (Yewers, 2016). Rather, the greater potential territory overlaps in 2011 may reflect environmental conditions with 2012 being much hotter and drier than 2011 (The Bureau of Meterology, 2013). In tree lizards, orange males are nomadic in drought years but not normal years (Moore et al., 1998; Paterson & Blouin‐Demers, 2018). However, we found no evidence that *C. decresii* color morphs responded differently to the different environmental conditions in the 2 years we sampled. Environmental factors, including aridity, strongly influence the relative frequency of *C. decresii *color morphs between populations, with a higher proportion of orange and lower proportion of yellow males in more arid environments (McLean, Stuart‐Fox, & Moussalli, 2015). Thus, environmental factors could influence the relative fitness of color morphs, despite similar space use.

The weak genetic differentiation we detected between morphs could be explained by assortative mating rather than space use. Assortative mating can reduce gene flow among morphs and under restricted conditions can lead to divergence and reproductive isolation (Jiang et al., 2013; Kirkpatrick & Ravigné, 2002). Indeed in general, color polymorphism can promote speciation (Hugall & Stuart‐Fox, 2012); however, ecological and social constraints on mate choice may mean that assortative mating is not always possible, despite greater genetic compatibility of like‐morph pairs (Pérez I De Lanuza et al., 2013; Pryke, 2010; Pryke & Griffith, 2009). Thus, assortative mating can contribute to the maintenance of polymorphism within a population in combination with other evolutionary processes such as frequency‐dependent selection (Bleay et al., 2007; Kokko, Griffith, & Pryke, 2014). We can only speculate if or how assortative mating is occurring in *C. decresii*. However, through captive breeding trials, in which expression of the male throat color polymorphism was induced in females with testosterone (Rankin & Stuart‐Fox, 2015), we know that all combinations of morph pairs produce viable offspring, including matings between the most genetically different orange‐yellow and gray morphs (Rankin et al., 2016). In these pairings, females did not have a choice of mate, so we cannot discount the role of nonrandom female mate choice (i.e., assortative mating). Furthermore, if there were any incompatibilities between morphs in certain mating pairs, the effects could be subtle due to the weak observed genetic differentiation. Therefore, we also cannot discount the role of postcopulatory cryptic female choice and/or postzygotic genetic incompatibilities resulting in differences in offspring viability and mortality as juveniles and adults (Pryke & Griffith, 2009).

## 5 CONCLUSIONS

The combined evidence of behavioral (Yewers et al., 2016) and endocrinological differences (Yewers et al., 2017), morph heritability (Rankin et al., 2016), and microsatellite genetic differentiation together suggest a genetic polymorphism in *C. decresii* with a suite of correlated phenotypic traits. These correlated traits are likely controlled by a shared genetic mechanism (e.g., supergene, regulatory gene/s with pleiotropic effects; Rankin et al., 2016) because both theory and empirical studies suggest color polymorphism and associated phenotypic differences (e.g., morphology, behavior, life history, endocrinology) are governed by few genes of major effect, preventing breakdown of co‐adapted traits by recombination (Sinervo & Svensson, 2002, reviewed in McKinnon & Pierotti, 2010). Elucidating genetic mechanisms maintaining the polymorphism would require detailed studies on genomic architecture. In terms of evolutionary processes maintaining the polymorphism in *C. decresii*, our data on space use and fine‐scale genetic structure suggest that the influence of spatial arrangement of morphs and nonrandom mating between morphs is likely to be minimal. Instead, other processes, such as frequency‐dependent selection, are likely to be more important in maintaining the polymorphism in this species.

## CONFLICT OF INTEREST

The authors declare that they have no competing interests.

## AUTHOR CONTRIBUTIONS

MY and DSF designed the research, MY performed the fieldwork. MY and CM performed the laboratory work and genetic analysis. MY analyzed and interpreted the data. MY wrote the manuscript. All authors edited, read, and approved the final manuscript.

## DATA ACCESSIBILITY

Data will be archived upon acceptance of the manuscript. The datasets used and/or analyzed during the current study are available from Dryad: https:doi.org/10.5061/dryad.qm0bd40
