# Supplementary material for: Space use and genetic structure do not maintain color polymorphism in a species with alternative behavioral strategies
Source: Ecol Evol. 2018 Dec 26;9(1):295–306. doi: 10.1002/ece3.4729 (PMC6342114; doi:10.1002/ece3.4729)
Supplement: Supplementary file 1 [file ECE3-9-295-s001.docx]

**Appendix**

**Table S1**: Genetic differentiation between colour morphs of *C. decresii* generated from multilocus genotypes (n=8 microsatellite loci) excluding estimated null alleles, with pairwise F_ST_ values and p-values.

| Comparison | F_ST_ | p |
| --- | --- | --- |
| Grey & Orange | 0.004 | 0.269 |
| Grey & Orange-yellow | 0.013 | 0.045 |
| Grey & Yellow | 0.004 | 0.270 |
| Orange & Orange-yellow | 0.003 | 0.899 |
| Orange & Yellow | 0.006 | 0.034 |
| Orange-yellow & Yellow | 0.002 | 0.551 |

No pairwise F_ST_ values remained significant after False Discovery Rate correction.

**Table S2**: Pairs of loci that show significant linkage disequilibria within morphs and across the population. The orange-yellow morph has been abbreviated to OY

| Pairs of Loci | Grey | Orange | OY | Yellow | All |
| --- | --- | --- | --- | --- | --- |
| Ctde08 & Ctde45 | **<0.001** | **<0.001** | **<0.001** | **0.002** | **<0.001** |
| Ctde12 & Ctde45 | **0.007** | 0.116 | 1 | 0.189 | 0.421 |
| Ctde05 & CP10 | **<0.001** | 1 | 1 | 0.188 | 0.028 |
| Ctde08 & CP10 | **0.002** | 1 | 1 | 0.193 | 0.053 |
| Ctde12 & CP10 | **<0.001** | 1 | 1 | 0.436 | **<0.001** |
| Ctde12 & Ctde21 | **0.102** | 1 | 0.306 | **<0.001** | 0.006 |

Significant p-values in bold remained significant after False Discovery Rate correction.

**Table S3:** Mantel test results for matrix correlations between pairwise geographic and genetic distances generated in GeneAlEx for each year sampled (sample size = N, correlation coefficients = r, p-values = p)

| Year | Morph | N | r | p |
| --- | --- | --- | --- | --- |
| 2011 | Grey | 10 | -0.04 | 0.435 |
|  | Orange | 11 | -0.007 | 0.486 |
|  | Orange-yellow | 16 | 0.025 | 0.38 |
|  | Yellow | 16 | 0.133 | 0.09 |
|  | Overall | 53 | 0.012 | 0.383 |
| 2012 | Grey | 14 | -0.047 | 0.352 |
|  | Orange | 10 | 0.2 | 0.107 |
|  | Orange-yellow | 17 | -0.027 | 0.445 |
|  | Yellow | 20 | -0.026 | 0.401 |
|  | Overall | 62 | -0.002 | 0.503 |


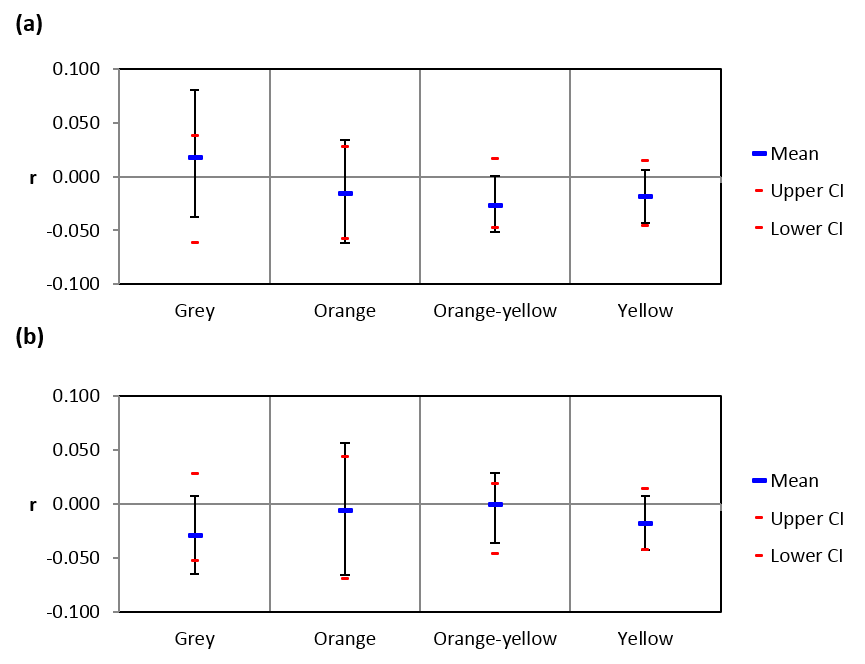


**Figure S1:** Mean relatedness (r coefficient; [Queller and Goodnight (1989)](#_ENREF_68)) of individuals within colour morphs in 2011 (**a**) and 2012 (**b**). Upper and lower 95% confidence interval values are represented by red marks. Coefficients are on a scale of 0 (not related) to +/-1 (clones or identical twins), with negative values resulting when two individuals differ from the population mean in different directions.
